# Hypoxia induces purinergic receptor signaling to disrupt endothelial barrier function

**DOI:** 10.3389/fphys.2022.1049698

**Published:** 2022-11-21

**Authors:** Somasundaram Raghavan, Masuma Akter Brishti, Daniel Mohr Collier, M. Dennis Leo

**Affiliations:** Department of Pharmaceutical Sciences, University of Tennessee Health Science Center, Memphis, TN, United States

**Keywords:** hypoxia, endothelial cells, purinergic receptor, blood-brain barrier, diabetes

## Abstract

Blood-brain-barrier permeability is regulated by endothelial junctional proteins and is vital in limiting access to and from the blood to the CNS. When stressed, several cells, including endothelial cells, can release nucleotides like ATP and ADP that signal through purinergic receptors on these cells to disrupt BBB permeability. While this process is primarily protective, unrestricted, uncontrolled barrier disruption during injury or inflammation can lead to serious neurological consequences. Purinergic receptors are broadly classified into two families: the P1 adenosine and P2 nucleotide receptors. The P2 receptors are further sub-classified into the P2XR ion channels and the P2YR GPCRs. While ATP mainly activates P2XRs, P2YRs have a broader range of ligand selectivity. The P2Y1R, essential for platelet function, is reportedly ubiquitous in its expression. Prior studies using gene knockout and specific antagonists have shown that these approaches have neuroprotective effects following occlusive stroke. Here we investigated the expression of P2Y1R in primary cultured brain endothelial cells and its relation to the maintenance of BBB function. Results show that following *in vitro* hypoxia and reoxygenation, P2Y1R expression is upregulated in both control and diabetic cells. At the same time, endothelial junctional markers, ZO-1 and VE-cadherin, were downregulated, and endothelial permeability increased. siRNA knockdown of P2Y1R and MRS 2500 effectively blocked this response. Thus, we show that P2Y1R signaling in endothelial cells leads to the downregulation of endothelial barrier function.

## Introduction

Purinergic signaling is essential for the maintenance of vascular function. Extracellular nucleotides were found to cause vasodilation nearly a century ago, but cellular signaling pathways related to these are surprisingly complex, and much about these signaling mediators remains unresolved. Purinergic receptors are broadly classified into two families. These are the G protein-coupled (GPCR) P1 adenosine receptors (A_1_, A_2A_, A_2B_, and A_3_) and the P2 nucleotide receptors. The latter are further subclassified into two groups, the P2XR ion channels, and the P2YR GPCRs. Currently, seven P2X and eight P2Y receptors have been identified, each with varying tissue distribution. P2XRs are activated mainly by adenosine 5′-trisphosphate (ATP), but P2YRs show a broader range of ligand selectivity with nucleotide phosphates. The native agonist for P2Y_1_R, P2Y_12_R, and P2Y_13_R is adenosine 5′‐diphosphate (ADP). The P2Y1R was first described in platelets and is essential for platelet function. ADP activates P2Y1R to stimulate platelet aggregation, and deficiency of P2Y1R increases bleeding time and reduces thrombosis. This effect has made the platelet P2Y_1_R an attractive target for antithrombotic therapy.

The blood-brain barrier (BBB), a critical feature of cerebral endothelial cells, tightly regulates access to and from the blood to the CNS and comprises “junctional” proteins that orchestrate the permeability of this barrier (Daniel and van Buul, 2013; Komarova et al., 2017; Duong and Vestweber, 2020). BBB function is altered in several diseases, increasing the incidence of stroke (Daniel and van Buul, 2013; Komarova et al., 2017; Duong and Vestweber, 2020). Extracellular nucleotides like ATP and ADP can signal through the purinergic receptors on the endothelium to influence the permeability of the BBB (Communi et al., 2000). Endothelial cells can release nucleotides in response to several physiological or pathological stimuli. (Gündüz et al., 2006; Gündüz, Hirche, Härtel, Rodewald, Schäfer, Pfitzer, Piper, Noll; Härtel et al., 2007). Studies have shown that activating P2Y receptors in the brain artery endothelial cell initiates Ca^2+^ and PKC signaling pathways (Sipos et al., 2000). In several studies using the middle cerebral artery occlusion (MCAO) model, investigators have shown that inhibition of the P2Y1 receptor offers distinct neuro-protective effects (Guzman and Gerevich, 2016). We wanted to observe the expression of the P2Y1 receptor in primary cultured mice cerebral artery endothelial cells and if it was involved in endothelial barrier function. We show that basal P2Y1 receptor expression is low even in diabetic cells and that following *in vitro* hypoxia/reoxygenation, there is an immediate upregulation of P2Y1R that signaled through protein kinase C to disrupt the endothelial barrier and increase endothelial permeability. MRS 2500, a P2Y1R selective antagonist, effectively reversed this barrier dysfunction. This study shows that a P2Y1R antagonist might effectively preserve endothelial function after injury.

## Materials and methods

### Cell culture

Mouse (C57BL/6 and db/db) primary brain microvascular endothelial cells were obtained from Cell Biologics Inc. (Cat# C57-6023 and MD-6023). Cells were cultured in Endothelial Cell Medium containing (5 ng/ml VEGF, 0.75 U/ml Heparin, 5 ng/ml EGF, 1 µg/ml Hydrocortisone, 10 mM L-Glutamine and 10,000 U penicillin (base), 10,000 U of streptomycin (base), and 25 µg of amphotericin B/ml as antimycotic in 0.85% saline) supplemented with 2% fetal bovine serum under standard cell culture conditions.

### Other reagents

P2Y1R (1:500 dilution, #PA5-77678) and Zonula occludens-1 (ZO-1, 1:500 dilution, #40-2200) antibodies were from Thermo Scientific Inc., VE-cadherin antibody (1:500 dilution, #MAB9381) was from Novus Biologicals, and Actin antibody (1:5000 dilution, MAB1501) was from Sigma-Aldrich. MRS 2500 was obtained from Tocris Biosciences, dissolved in water, and used at a final concentration of 100 nM. Xestospongin C (Xes-C) and bisindolylmaleimide II (BIM II) were obtained from Sigma-Aldrich, dissolved in DMSO, and used at a final concentration of 10 µM each. P2Y1R siRNA was purchased from ThermoScientific Inc., and used at a final concentration of 10 µg per culture plate. siRNA was transfected into the cells using Lipofectamine 3000 (ThermoScientific Inc.) 24 h prior to subjecting cells to H/RO. Disodium-ADP salt was from MP Biomedicals LLC and was used at final concentrations, as indicated in the results.

### Induction of *in vitro* hypoxia and reoxygenation

The oxygen-glucose deprivation/reoxygenation model was used for *in vitro* studies. This technique is referred to throughout the manuscript as hypoxia (H)/reoxygenation (RO). Briefly, endothelial cell culture media was replaced with aglycemic DMEM (Catalog#: 11966025, ThermoScientific Inc.) and placed in a hypoxia incubator chamber from Stemcell Technologies. The chamber was first purged with 1% O_2_, 10% CO_2,_ and 89% N_2_ gas mixture for 6 min and then sealed and held at a temperature of 37°C for 90 min. Following this, the cell cultures were removed, the media was replaced with regular endothelial culture media, and the cultures were returned to a standard cell culture incubator. Culture plates were harvested at the required time points. All inhibitors, MRS2500, Xes-C, and BIM II, were added at the start of reoxygenation. For P2Y1R knockdown experiments, siRNA was first transfected into the cells 24 h before the H/RO protocol.

### Endothelial barrier permeability assay

For this assay, we used the endothelial transwell permeability assay kit (Cell Biologics, Inc., Chicago, IL) as per the manufacturer’s instructions. Briefly, control and diabetic endothelial cells were seeded on the 6.5 mm transwell insert membrane. The inserts were then transferred to the cell culture well containing growth media. The plates were incubated for 2–4 days till confluence was observed. The EC cultures were then maintained as normoxic or subject to *in vitro* hypoxia/RO. 24 h after normoxia or RO, as indicated earlier. After the treatment period, the media from the lower chamber of the well was collected and added to wells of a regular ELISA plate. The provided TMB substrate was added to each well, incubated for 10 min at room temperature and the stop solution was added. Spectrophotometric readings were done at 450 nm using a plate reader. Data were normalized to normoxic controls and expressed as fold change.

### Surface biotinylation

Surface biotinylation was performed as we have done previously for intact arteries and endothelial cells (Leo et al., 2021; Raghavan and Leo, 2022). Briefly, live endothelial cells were the first subject to *in vitro* hypoxia for 90 min, followed by 24 h reoxygenation. After this period, cells were washed with warm phosphate-buffered saline (PBS), and the plates were then placed on ice for 30 min to inhibit all protein trafficking. Cell-impermeable biotinylation reagents were dissolved in cold-PBS are then added to the cultures and were allowed to incubate at 4°C with gentle shaking for an additional 30 min. At this end, the biotinylation reagents were removed by washing with PBS, and the reaction was quenched with 100 mM glycine (in PBS). Protein lysates were collected, and protein concentration was estimated by Amido black method. An equal concentration of protein from each group was passed through an avidin bead column to separate the biotinylated surface protein (henceforth denoted as “S”) and the non-biotinylated intracellular fraction (hereafter designated as “I”). Each sample was run as adjacent lanes on an SDS-gel as “S” or “I” fractions. Analysis was by semi-quantitative comparison between the surface and intracellular band intensities and was expressed as % of total protein.

### Western blotting

Western blotting to study protein expression was done following standard protocols. Following the avidin-pulldown and separation of cell lysates into the surface and intracellular fractions, Laemmeli buffer was added to the lysates and boiled for 4 min. Samples were then separated on 8% SDS-polyacrylamide gels and transferred onto nitrocellulose membranes. Membranes were first blocked with 5% non-fat milk for 1 h and then incubated with primary antibodies overnight at 4°C. Membranes were then washed with PBS/Tween-20 and incubated with horseradish peroxidase-conjugated secondary antibodies for 1 h at room temperature. Blots were physically cut to allow for the probing of two different proteins to limit the signal loss from stripping. Where unavoidable, blots were stripped with Stripping buffer (Thermo Scientific Inc.) for 10 min, re-blocked with 5% non-fat milk, and then processed with another primary/secondary antibody combination. Protein bands were imaged using a ChemiDoc gel imaging system and quantified using Quantity One software (Biorad).

### Statistical analysis

Statistical analysis was performed using OriginLab and GraphPad InStat software. Data are shown as mean ± SE and expressed as fold change compared to respective normoxia band intensities. Student’s *t*-test, Mann–Whitney *U* test and ANOVA with Bonferroni’s post hoc test for multiple group comparisons were used where appropriate. *n* values are the number of experiments. *p* < 0.05 was considered significant.

## Results

### Endothelial P2Y1 receptors are upregulated after hypoxic injury

**FIGURE 1:**
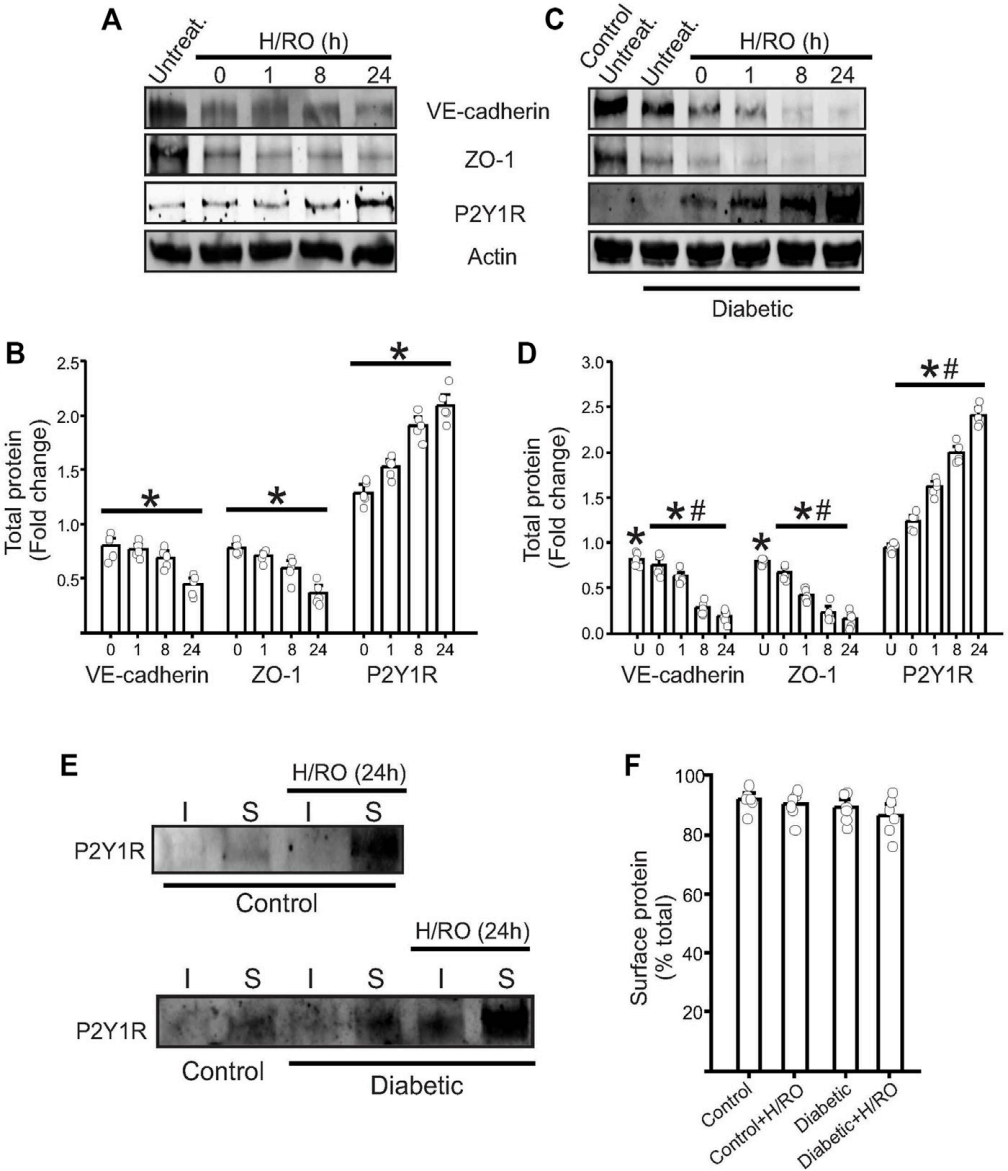
Upregulation of endothelial P2Y1 receptor post-H/RO decreases junctional protein expression. Representative Western blots showing the expression of VE-cadherin, ZO-1, P2Y1 receptor and actin in **(A)**, control and **(C)**, diabetic endothelial cells. **(B,D)** Bar graphs for the same presented fold change in total protein and as mean ± SE. *n* = 5 for each, * indicates *p* < 0.05 vs. normoxic control, # indicates *p* < 0.05 vs. untreated normoxic diabetic (U in bar chart). **(E)** Representative Western blots after surface biotinylation in control and diabetic cells showing P2Y1R expression in surface (S) and intracellular (I) fractions with or without H/RO (24 h). **(F)** Mean data showing the surface expression of the protein alone expressed as % total protein.

To better understand the role of P2Y1 receptors in endothelial cells, we first performed Western blotting in control and diabetic cells. Results showed that under basal conditions, control and diabetic endothelial cells had minimal protein expression of P2Y1R (Figures 1A–D). This suggested that in the brain microvasculature, P2Y1R only has a minor basal role in endothelial signaling pathways. Next, in a series of experiments, we subjected our control and diabetic cells to hypoxia for 90 min, followed by re-oxygenation for specific time points. In cells exposed to hypoxia, there was an immediate increase in P2Y1R expression following hypoxia, and in control cells, the receptor levels gradually increased over time and peaked at 24 h post-H/RO (Figures 1A,B). Diabetic endothelial cells also showed similar trends in P2Y1R expression (Figures 1C,D). However, at 24 h post-H/RO, P2Y1R expression was significantly greater than the equivalent time point in control (Figures 1C,D). This result indicated that following hypoxia and reoxygenation, endothelial cell P2Y1R expression gradually and substantially increases over time, with a more significant increase observed in diabetic endothelial cells.

### Endothelial junctional proteins are downregulated after hypoxic injury

To understand what consequences any P2Y1R upregulation might have on endothelial function, we next looked at endothelial barrier function. To analyze this, we probed for the expression of endothelial VE-Cadherin and ZO-1, which represent adherens and tight junctions, respectively. In control cells, there was an immediate and significant decrease in both proteins after hypoxia exposure. This expression further decreased over time during RO to peak at ∼70% lower than normoxic conditions (Figures 1A,B). In normoxic diabetic cells, VE-cadherin and ZO-1 expression was significantly lower than in normoxic controls (Figures 1C,D). These expression levels then decreased post-H/RO gradually to ∼90% lesser at 24 h post-H/RO (Figures 1C,D). These results indicate that control and diabetic cells exposed to H/RO have a significant loss in vital junctional proteins, which is much more severe in diabetic cells.

### Newly synthesized P2Y1 receptors translocate to the plasma membrane

We next investigated the cellular localization of P2Y1 receptors. Previous studies have shown that P2Y1 responds to extracellular ADP and is located at the plasma membrane. We performed surface biotinylation on intact normoxia and 24 h post-H/RO endothelial cells to analyze the cellular localization of P2Y1R. Western blotting of avidin-separated protein extracts confirmed that most of the P2Y1R is located at the cell surface in both control and diabetic cells (Figures 1E,F). This indicates that new P2Y1R is trafficked to the cell surface immediately following protein synthesis and initiates downstream signaling.

### Junctional protein degradation is directly linked to P2Y1R activation

**FIGURE 2:**
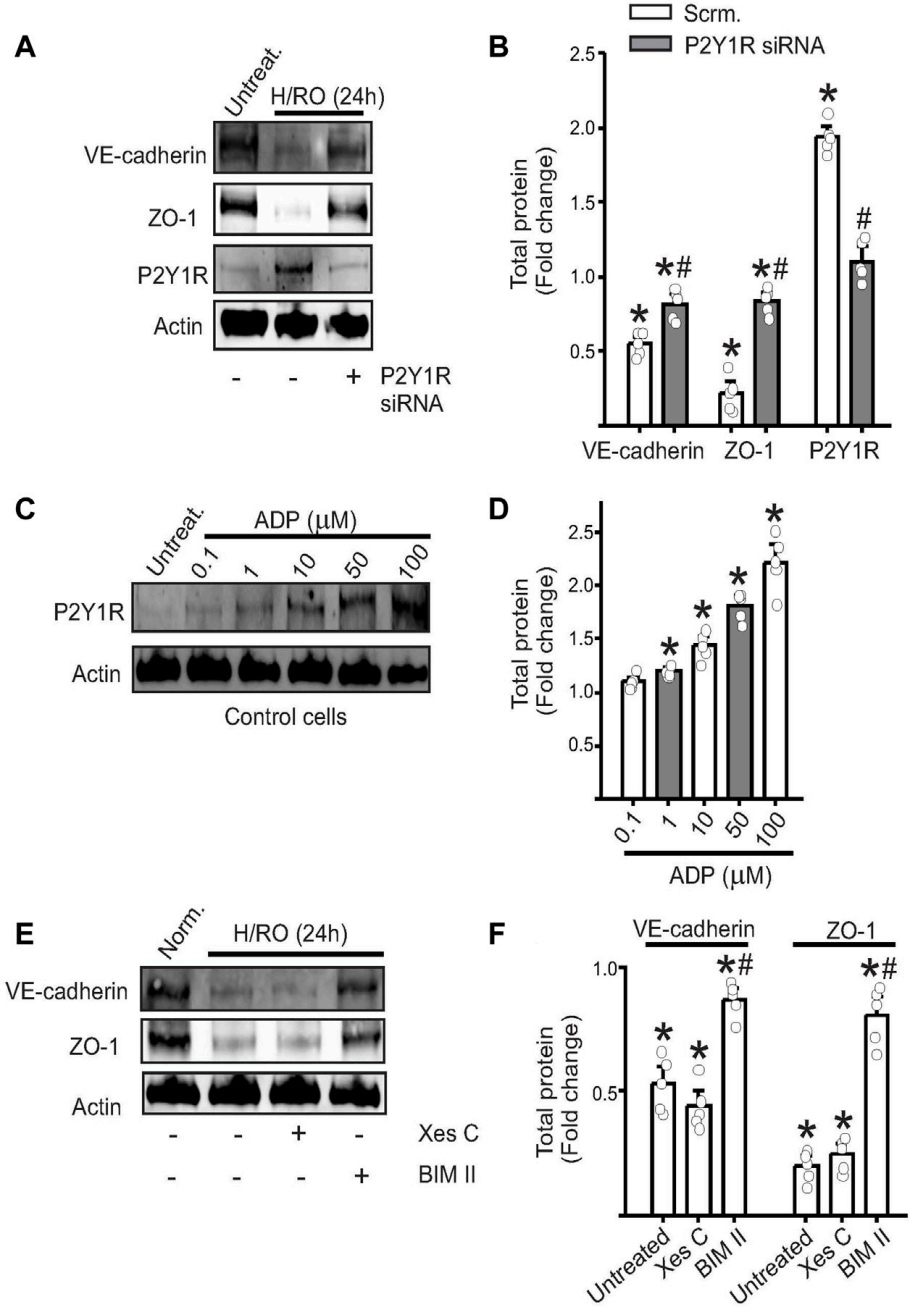
P2Y1 receptor signaling decreases endothelial junctional protein expression. Representative Western blots from control cell lysates showing the expression of VE-cadherin, ZO-1, P2Y1 receptor and actin in **(A)**, with or without H/RO and P2Y1R siRNA and **(B)**, bar graph for the same showing fold change in total protein. Mean ± SE. *n* = 5 for each, * indicates *p* < 0.05 vs. normoxic scrambled or no-treatment controls, # indicates *p* < 0.05 vs. H/RO (24 h) treatment. **(C)** Representative Western blots from control cell lysates showing the expression of P2Y1R after addition of ADP to the extracellular media at specific concentrations. **(D)** Mean data. *n* = 5 for each, * indicates *p* < 0.05 vs. untreated controls. **(E)** Representative Western blots from control cell lysates showing the expression of proteins in control cells with or without H/RO and Xestospongin C (Xes C) or bisindolylmaleimide II (BIM II) treatment. **(F)** Bar graphs of mean protein expression post-H/RO presented as fold change. n = 5 for each, * indicates *p* < 0.05 vs. normoxic controls, # indicates *p* < 0.05 vs. untreated H/RO.

To verify if P2Y1R downstream signaling initiated the degradation of VE-cadherin and ZO-1, we transfected endothelial cells with P2Y1R siRNA and then maintained them at normoxia or subjected them to the H/RO protocol. Western blotting revealed that in the presence of P2Y1R siRNA, P2Y1R protein expression remained at basal levels even after H/RO (Figures 2A,B). Similarly, the expression of VE-cadherin and ZO-1 also partially recovered post-H/RO after the knockdown of P2Y1R (Figures 2A,B). These results indicate that the degradation of junctional proteins was likely a result of P2Y1R protein and signaling upregulation post-H/RO.

### Endothelial cell-released ADP upregulates P2Y1R expression

The agonist for P2Y1R receptors is extracellular ADP, but the culture media is unlikely to have significant levels of ADP. Since most P2Y1R were located at the cell surface, it was likely that they were being activated by ADP released by the stressed endothelial cells themselves. Given the technical limitations of multiple and accurate ADP measurements in the extracellular media, we chose to test this hypothesis in control cells and by adding defined ADP concentrations. In confluent control endothelial cells maintained under normoxic conditions, we added ADP at final concentrations of 0.1, 1, 10, 50, and 100 µM for 1 hour. Results indicate that increasing concentrations of ADP also increased P2Y1R expression, with the most significant upregulation recorded for 100 µM ADP (Figures 2C,D). This result suggests that following hypoxia, there is likely a significant release of ADP from endothelial cells. This extracellular ADP not only triggers activation of P2Y1R signaling but also induces upregulation of the receptor itself.

### P2Y1R signaling leads to protein kinase C activation

We then wanted to investigate which downstream mediator of P2Y1R signaling caused endothelial junctional protein degradation. P2Y1R signaling activates endoplasmic reticulum Ca^2+^ release and protein kinase C (PKC). We used xestospongin-C (Xes-C) and BIM-II, inhibitors of ER Ca^2+^ release and PKC, respectively, to block these pathways. The inhibitors were added at the start of reoxygenation and were allowed to remain for the remainder of the experiment. Western blotting on lysates revealed that Xes-C did not affect either VE-cadherin or ZO-1 expression (Figures 2E,F). However, in the presence of BIM-II, the PKC inhibitor, both junctional proteins’ expression was restored to normoxic levels (Figures 2E,F). These results indicate that P2Y1R activation leads to downstream PKC signaling, which affects the stability of the proteins involved in barrier function.

### P2Y1R antagonist reverses junctional protein loss after hypoxia

**FIGURE 3:**
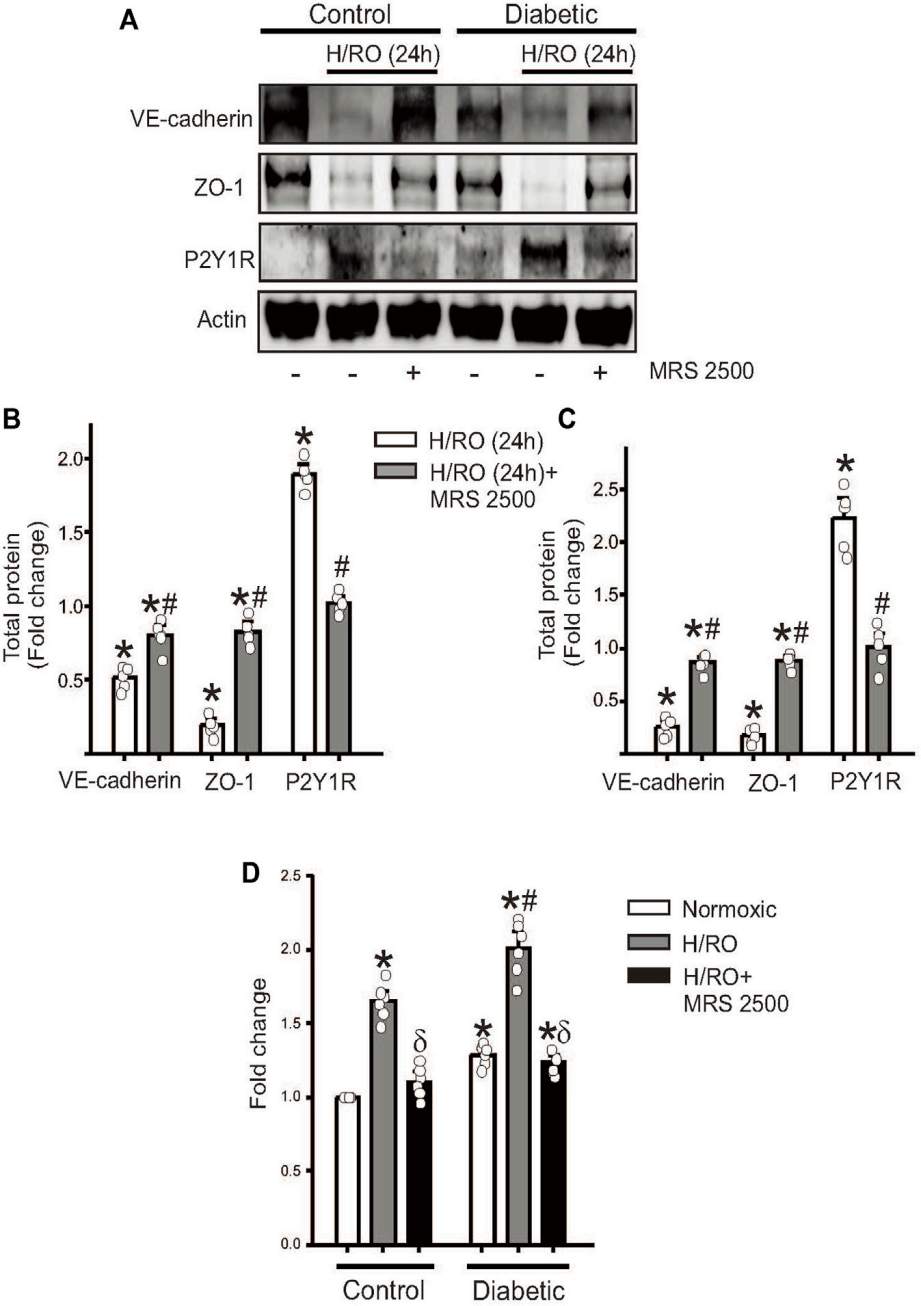
P2Y1R antagonist, MRS 2500, rescues endothelial function after H/RO. Representative Western blots showing the expression of VE-cadherin, ZO-1, P2Y1 receptor and actin in **(A)**, control and diabetic endothelial cells with or without H/RO and MRS 2500 treatment. Bar graphs for protein expression in control, **(B)**, and diabetic cells, **(C)**, presented as fold change in total protein and mean ± SE. *n* = 5 for each, * indicates *p* < 0.05 vs. untreated control, # indicates *p* < 0.05 vs. respective H/RO (24 h) treatment. **(D)**. Bar graph showing fold change in endothelial transwell permeability in normoxic, after H/RO (24 h) alone and H/RO (24 h) +MRS 2500 treated cells. *n* = 6 for each. * indicates *p* < 0.05 vs. normoxic control, # indicates *p* < 0.05 vs. normoxic diabetic and *δ* indicates *p* < 0.05 vs. respective H/RO (24 h).

We then sought to study the effect of a well-known P2Y1R antagonist, MRS 2500, on hypoxia-induced P2Y1R signaling in both control and diabetic cells. MRS 2500 was added during reoxygenation, and 24 h after addition, protein lysates were collected for Western blotting. Results from control and diabetic cells subject to H/RO showed that adding the P2Y1R antagonist restored VE-cadherin and ZO-1 expression to approximately similar levels to normoxic controls (Figures 3A–C). Interestingly, the antagonist decreased the expression of P2Y1R itself, suggesting that receptor blockade possibly induces degradation of the protein (Figures 3A–C). Secondly, we performed an endothelial permeability assay to assess the functional relevance of P2Y1R inhibition. In control cells, H/RO significantly increased endothelial permeability after 24 h, which was completely reversed by the addition of MRS 2500 at the start of RO (Figure 3D). In diabetic cells, however, untreated cells already showed increased permeability compared to untreated controls (Figure 3D). After H/RO, permeability increased further compared to the similar time point in controls (Figure 3D). When H/RO subject diabetic cells were treated with MRS 2500, cell permeability decreased but only to normoxic cells and did not have any additional benefit. These results suggest that MRS2500, a P2Y1R antagonist, prevents junctional protein degradation and permeability dysfunction post-H/RO in control endothelial cells. Although a similar effect was observed in diabetic cells, the compound could not reset the basal expression of proteins already altered in diabetic cells.

Together, these results indicate that in an *in vitro* hypoxia model, MRS 2500, a P2Y1R antagonist, effectively limits damage to the endothelial barrier by decreasing P2Y1R-dependent, PKC-mediated degradation of junctional proteins.

## Discussion

Here, we investigated the hypothesis that endothelial cell-P2Y1 receptor signaling following hypoxic stress leads to endothelial barrier dysfunction and hyperpermeability. A P2Y1R antagonist can prevent these changes to protect endothelial barrier function. Our data here showed that in both control and diabetic mouse brain microvascular endothelial cells, basal P2Y1R protein expression is very low. Following a well-established method of inducing hypoxic stress in cell culture, we found that post-H/RO, the expression of P2Y1R gradually increases over time to peak at 24 h, the furthest time-point studied here. Diabetic cells showed a more significant increase in P2Y1 expression at 24 h post-H/RO compared to the same time point in control cells. Interestingly, the expression of two critical endothelial junctional proteins, VE-cadherin and ZO-1, proportionately decreased within the same period. Further analysis revealed that most of the newly synthesized P2Y1R was translocated to the plasma membrane and that P2Y1R primarily signaled through PKC and not through ER-Ca^2+^ release to induce degradation of the junctional proteins. The addition of extracellular ADP to control normoxic cells upregulated P2Y1R expression. Finally, we also show that the P2Y1R antagonist, MRS 2500, when added during RO, prevented the loss of VE-cadherin and ZO-1, induced degradation of the excess P2Y1R, and restored endothelial cell permeability to normoxic levels.

The blood-brain barrier (BBB), a crucial feature of the CNS microvasculature, tightly controls the movement of nutrients, metabolites, and cells from the blood to and from the brain (Daniel and van Buul, 2013; Komarova et al., 2017; Duong and Vestweber, 2020). BBB function is altered in several diseases, including hypertension, diabetes, acute and chronic inflammation, and cancer, and can lead to an increase in the incidence of stroke (Daniel and van Buul, 2013; Komarova et al., 2017; Duong and Vestweber, 2020). The overall function of this barrier is controlled by critical proteins called “junctional proteins”, which are expressed by the endothelial cells that line the vasculature of the CNS. The BBB is sub-organized into three types of junctions, tight junctions (TJ), adherens junctions (AJ), and gap junctions (GJ). While Zona occludens-1 (ZO-1) is a frequently studied TJ protein, VE-cadherin is a commonly described protein for AJ function (Daniel and van Buul, 2013; Komarova et al., 2017; Duong and Vestweber, 2020).

Extracellular nucleotides like adenosine triphosphate (ATP) and its metabolites, adenosine diphosphate (ADP), and adenosine, can influence the integrity of the BBB by signaling through the purinergic receptors of the endothelium (Communi et al., 2000). In the vasculature, the primary nucleotide sources are platelets, erythrocytes, and endothelial cells (Aslam et al., 2021). Degranulation of platelets can cause plasma levels of ATP to reach 50 µM or higher (Gündüz et al., 2006; Härtel et al., 2007; Aslam et al., 2021). Endothelial cells can release nucleotides in response to several physiological or pathological stimuli. For example, shear stress, inflammatory mediators, hypoxia, change in osmolarity, and blood glucose elevations are all known to induce endothelial nucleotide release, and it is predicted that local concentrations of nucleotides could be in the order of several micromoles (Gündüz et al., 2006; Gündüz, Hirche, Härtel, Rodewald, Schäfer, Pfitzer, Piper, Noll; Härtel et al., 2007). Early studies on the effect of nucleotides have shown that ATP, ADP, and UTP evoke Ca^2+^ responses and suggested that P2Y receptors are present in the brain artery endothelial cells (Sipos et al., 2000). Much of what is known about P2Y1R are investigations into its role in NO-mediated relaxation in endothelial cells and neuromodulation after stroke or degenerative diseases (Aslam et al., 2021). *P2RY1* knockout mice are physically normal but are susceptible to lung infections (Fabre et al., 1999; Geary et al., 2005). Unsurprisingly, they are resistant to ADP-induced thrombin formation (Fabre et al., 1999). Double knockout of *P2RY1* and *apoE* show reduced atherosclerotic lesions traced to signaling originating from vascular P2Y1R (Hechler et al., 2008). In basilar arteries from rats following subarachnoid hemorrhage (SAH), the expression of P2Y1R increased 5 days after and subsided by 7 days after SAH (Carpenter et al., 2001). In *P2RY1* knockouts, leukocyte transendothelial migration was reduced, suggesting that P2Y1R could potentiate endothelial barrier breakdown and cause hyperpermeability (Zerr et al., 2011). In HUVECS, P2Y1R-induced Ca^2+^-dependent activation of Rac1 decreased hyperpermeability caused by thrombin (Soulet et al., 2005). Conversely, in a mouse model of traumatic brain injury, the P2Y1R agonist 2-methylthioadenosine 5′diphosphate (2MeSADP) delayed the development of cerebral edema (Talley Watts et al., 2013).

In our brief study, we found that primary cultured brain microvascular endothelial cells have a very low basal expression of P2Y1R and almost immediately increased after the cells were exposed to *in vitro* hypoxia. The increase in P2Y1R expression and signaling then appeared to trigger the downregulation of endothelial junctional proteins. P2Y1R is a Gq-linked GPCR that initiates signaling *via* both PKC and the release of intracellular Ca^2+^. Several PKC isoforms are known to disrupt endothelial permeability (Ferro et al., 2000; Siflinger-Birnboim and Johnson, 2003; Li et al., 2004; Tang et al., 2018), so it was unsurprising that the PKC inhibitor, BIM II, effectively prevented endothelial barrier dysfunction. Although hypoxia and RO could have triggered PKC through other pathways, the fact that P2Y1R siRNA or MRS 2500 were also effective suggested that signaling originating from P2Y1R is an essential component in PKC activation post-H/RO. In a previous report (Raghavan et al., 2021), we have shown that junctional protein association with Rab5 increases internalization and degradation of the proteins. P2Y1R-induced PKC signaling possibly triggers a similar effect post-H/RO. In animal models following middle cerebral artery occlusion (MCAO), either the P2Y1R knockout or the antagonists, MRS 2500 and MRS 2179 were effective in reversing the deficit in fear-based learning, recognition memory, spatial memory, and working memory (Guzman and Gerevich, 2016). P2Y1R antagonists likely offer neuroprotection in neurons and astrocytes by preventing ATP, glutamate, and inflammatory cytokine release (Förster and Reiser, 2015). Endothelial cells also actively respond to inflammatory or hypoxic stress by releasing several mediators. While it is likely that the downregulation of junctional proteins is a defensive mechanism to support immune cell transendothelial migration, it is possible that this process also allows several undesirable molecules that might appear post-H/RO unfettered access into the CNS. Hence, a P2Y1R antagonist could offer protection at multiple levels after a significant CNS stress event, from preventing endothelial barrier dysfunction to preventing neuronal activation.

An interesting observation in this study was with the agonist that likely activates P2Y1R, which our results show could be endothelial cell-released ADP. A limitation here is that we did not directly test ADP levels post-H/RO. It is likely that the process of ADP release from endothelial cells is highly dynamic and will require more sensitive ATP/ADP flux analyzers to determine in detail. The mechanism and the identity of the endothelial cell ADP release channel activated post-H/RO is an exciting area that will need to be explored in the future.

In conclusion, we have shown that *in vitro* hypoxia/reoxygenation injury triggered endothelial cell P2Y1R upregulation, contributing to endothelial junctional protein degradation and increased endothelial permeability. A P2Y1R antagonist, MRS 2500, added immediately after the hypoxic injury effectively blocked the receptor and improved endothelial barrier permeability.

## Data Availability

The original contributions presented in the study are included in the article/Supplementary Material, further inquiries can be directed to the corresponding author.
