# Supplementary figures and images for: Hypoxia induces purinergic receptor signaling to disrupt endothelial barrier function
Source: Front Physiol. 2022 Nov 21;13:1049698. doi: 10.3389/fphys.2022.1049698 (PMC9720161; doi:10.3389/fphys.2022.1049698)

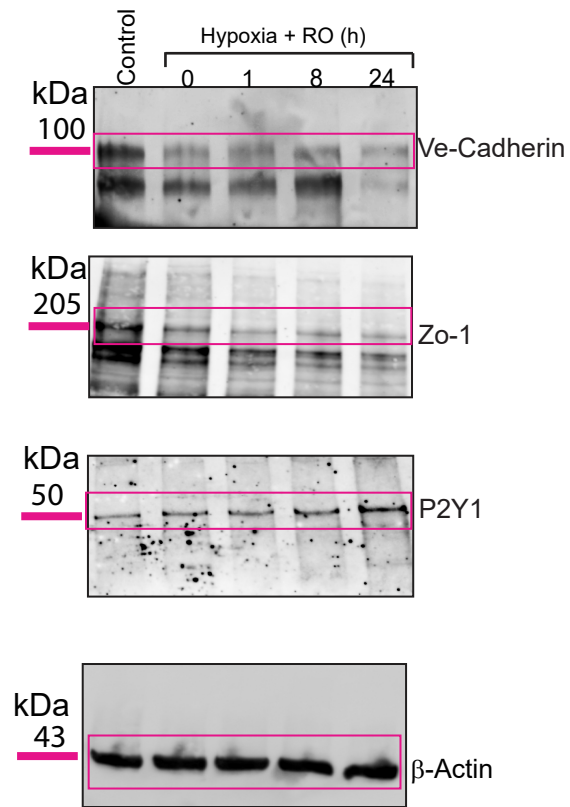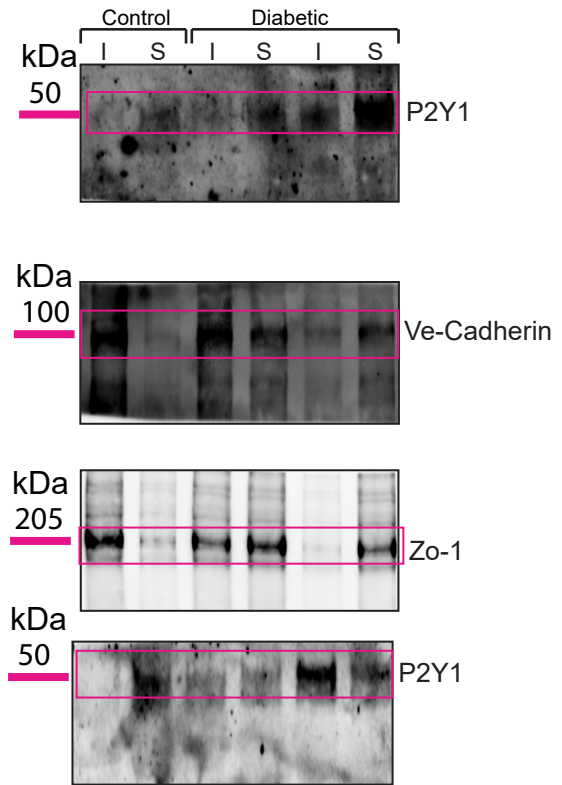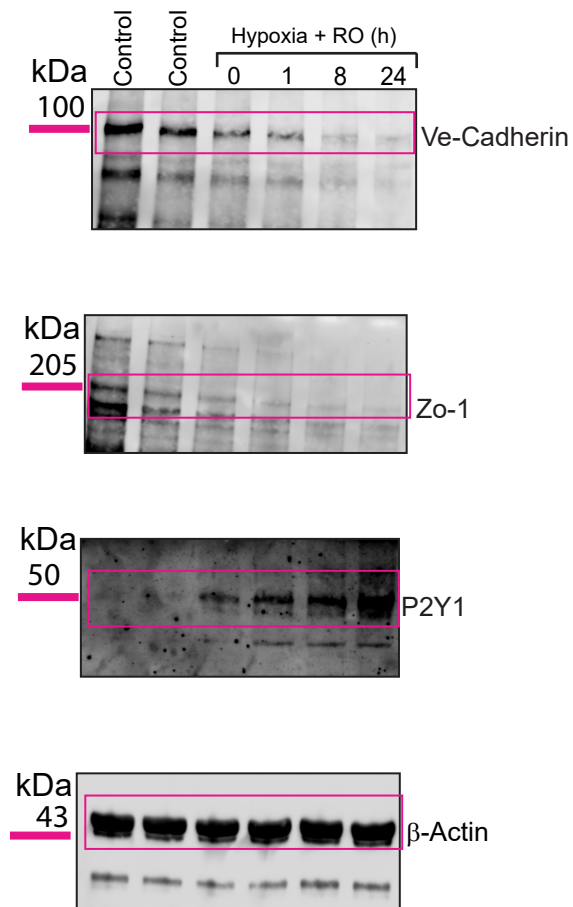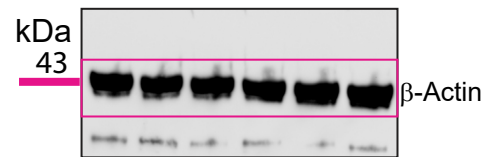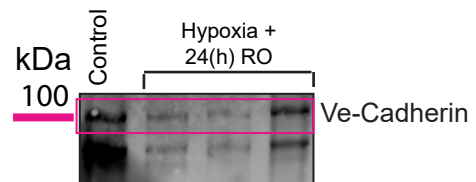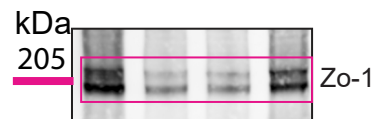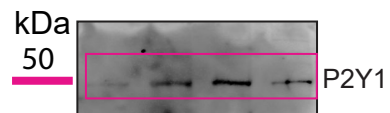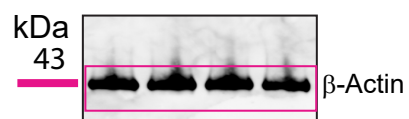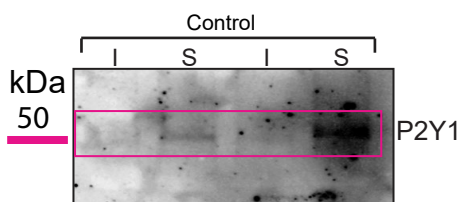

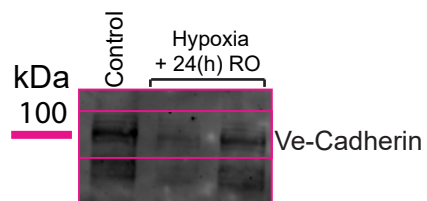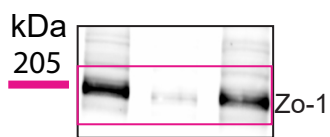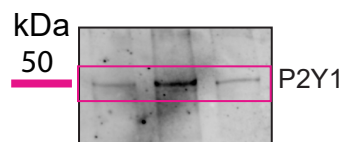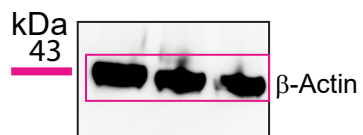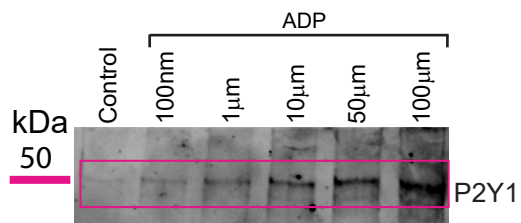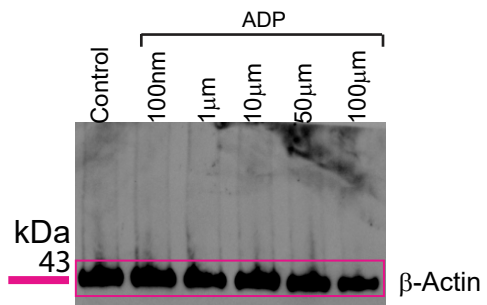

Supplement: Supplementary file 1 [file DataSheet1.PDF]
